# Genetic Variations Controlling Regulatory T Cell Development and Activity in Mouse Models of Lupus-Like Autoimmunity

**DOI:** 10.3389/fimmu.2022.887489

**Published:** 2022-05-26

**Authors:** Tracoyia Roach, Laurence Morel

**Affiliations:** Department of Pathology, Immunology, and Laboratory Medicine, University of Florida, Gainesville, FL, United States

**Keywords:** regulatory T cells, Foxp3, autoimmunity, lupus, genetics

## Abstract

Immune homeostasis is a constant balancing act between effector T cells and regulatory T cells defined by *Foxp3* expression, the transcription factor that drives their differentiation and immunosuppressive activity. Immune homeostasis is altered when Treg cells are not generated or maintained in sufficient numbers. Treg cells rendered unstable by loss of *Foxp3* expression, known as ex-Treg cells, gain pro-inflammatory functions. Treg cells may also become dysfunctional and lose their suppressive capabilities. These alterations can cause an imbalance between effector and regulatory subsets, which may ultimately lead to autoimmunity. This review discusses recent studies that identified genetic factors that maintain Treg cell stability as well as preserve their suppressive function. We focus on studies associated with systemic lupus erythematosus and highlight their findings in the context of potential therapeutic gene targeting in Treg cells to reverse the phenotypic changes and functional dysregulation inducing autoimmunity.

## Introduction

Regulatory T cells maintain immune homeostasis and prevent autoimmune diseases by limiting the responses of proinflammatory and autoimmune T cells. Several subsets of Treg cells have been characterized, among which the classical Foxp3^+^ CD4^+^ T cells, referred to here as Treg cells, play an essential role. The mechanisms by which these cells maintain immune homeostasis involve inhibitory cytokines, cytolysis, and metabolic disruption of effector T (Teff) cells (1). Treg cells are defined by the stable expression of *Foxp3, a* forkhead/winged helix transcription factor, and high levels of the high affinity interleukin-2 receptor (IL-2R) α chain (CD25) on their surface (2), which are the main genes required for Treg cell development, maintenance, and function (3). There are two major types of Treg cells: thymus Tregs (tTregs) that develop in the thymus, and peripheral Treg (pTregs) cells that are generated in peripheral sites. In addition, studies have been conducted on induced Treg (iTregs) cells that are induced *in vitro* by T cell receptor (TCR) activation in the presence of TGFβ (4). Treg cell stability, i.e. the maintenance of their transcriptional program, is indispensable for the preservation of their function. Furthermore, unstable or “ex-Treg” cells induce inflammation not just by a lack of suppression but also in a direct manner by secreting inflammatory cytokines (5).

## Defective Number or Frequency of Tregs Lead to Autoimmune Diseases

Numerical and/or functional Treg anomalies contribute to autoimmune diseases such as type 1 diabetes (6), rheumatoid arthritis (7), and systemic lupus erythematosus (SLE) (8). The absolute number of circulating Treg cells is decreased in SLE patients with active disease as compared to healthy controls (9–13). The number of Treg cells was shown to have a strong inverse correlation with SLEDAI (Systemic Lupus Erythematosus Disease Activity Index) scores, showing the lower numbers of Treg cells corresponding to increased disease severity (11, 12). Controversially, other studies have reported increased (14–16) or similar (17, 18) Treg cell numbers in SLE patients as compared to healthy controls. The discrepancy between these studies has been attributed to different definitions and gating strategies for Treg cells, including the fact that expression of Foxp3 alone is not a reliable marker for human Treg cells, further complicating analyses of their function and stability (12, 19). Variations in the treatment regimen with immunosuppressive drugs may also contribute to the large variations in relative Treg cell frequencies in SLE patients. Dysfunctional Treg cells have also been reported in SLE patients (8), including the expansion of a Treg population with a low CD25 expression (20).

This review discusses recent studies that have identified intrinsic genetic factors maintaining Treg cell stability as well as preserving their suppressive function. We focus on studies associated with systemic lupus erythematosus pathogenesis, or with a lupus-like phenotype, and we highlight their findings in the context of potential therapeutic gene targeting in Treg cells to reverse the phenotypic changes and functional dysregulation inducing systemic autoimmunity. The many studies that have reported gene targeting affecting Treg cells in other autoimmune diseases such as arthritis, uveitis or experimental autoimmune encephalomyelitis are not included in this review. Additionally, other studies that have reported deletions of specific genes in other cell types, such as dendritic cells, that affect Treg cell development and function are also not mentioned in this review.

## Single Gene Determinants of Treg Cell Homeostasis

Scrufy mice do not produce Treg cells due to a mutation in *Foxp3*, causing them to develop a severe inflammatory disease with autoimmune components, including lupus-like manifestations (21). A large number of studies have now defined *Foxp3* as the master regulator of Treg cell differentiation and functions (3). A recent study has shown that *Foxp3* sustained expression is also necessary to maintain Treg functions once they have differentiated (22). Reverse genetic approaches have identified several genes that control Treg cell number, stability and/or functions through *Foxp3* expression, and whose deficiency or overexpression lead to autoimmunity or lupus-like manifestations.

### Negative Regulators of *Foxp3* Expression

The AP1 transcription complex is comprised of a network of heterodimers formed by proteins of the Jun, Fos, ATF, and MAF families. Fos/Jun dimers promote the expression of *Foxp3* through direct binding to its promoter in response to TCR signaling (23). Within this transcription complex, Fos-like 2 (*Fosl2*) inhibits Treg development in a cell-intrinsic manner (24). *Fosl2* transgenic mice develop spontaneous autoimmunity and systemic inflammation with disease phenotypes resembling that of Treg-deficient IPEX patients and scurfy mice. On the other hand, mice lacking *Fosl2* in CD4^+^ T cells display less severe disease phenotypes. Mechanistically, Fosl2 interrupts Treg development by repressing the expression of *Foxp3* as well as that of other genes involved in Treg differentiation or function (24).

NFIL3 (Nuclear factor Interleukin 3 regulated, also known as E4 binding protein 4, E4BP4) represses numerous genes and regulates diverse biological processes (25, 26). In the immune system, NFIL3/E4BP4 has a vital role for many cell types including Th1, Th2, NKT and Treg cells by regulating the plasticity of cytokine production (27, 28). Treg cells are the T cell subset with the lowest *Nfil3* expression, and its overexpression attenuated the suppressive ability and stability of these cells (29). Not only does NFIL3 binds directly to the *Foxp3* promoter reducing *Foxp3* expression, but it also downregulates the promoter activity of Treg hallmark genes such as *Icos*, *Tnfrsf18*, *Ctla4*, and *Il2ra*, in both Foxp3-independent and dependent pathways (29). Accordingly, *Nfl3*-deficiency in T cells increased *Foxp3* expression, but decreased the frequency of *Foxp3*-expressing follicular regulatory T (Tfr) cells, resulting in an expansion of follicular helper T (Tfh) cells and the production of autoantibodies (30). Tfr cells are a specialized subsets of tissue Treg cells that work to constrain the activity of Tfh cells and germinal center (GC) B cells with whom they share the Bcl6 transcription factor (31). A decreased relative frequency of Tfr cells has been correlated with disease activity in SLE patients (32). NFIL3 expression was increased and its phosphorylation was decreased in CD4^+^ T cells from patients with SLE with a positive correlation to disease activity (30). These alterations were associated with the characteristic expansion of Tfh cells in SLE. It would be of great interest to follow up this study with an analysis of the impact that NFIL3 increased expression and decreased phosphorylation has on Treg and Tfr cell numbers and functions in SLE.

### Positive Regulators of Foxp3 Expression

NF-kβ is one of the multi-molecular complexes that interacts with Foxp3 to control Treg cell transcriptional programs and biology. c-Rel, one of its subunits activated by TCR signaling, supports tTreg development and *Foxp3* expression by binding to its promoter and one of its regulatory non-coding sequences (CNS3) (33). NF-kβ maintains the stability of mature Treg cells by preventing them from converting into effector-like T cells through mechanisms involving IKK*α* and IKKβ kinases, which are upstream activators of the NF-kβ pathway (34, 35). Foxp3 forms a complex with Rel-A, one of the most abundant NF-kβ subunits in conventional T cells, and with other transcription factors including Helios and p300, leading to its full functionality as a transcriptional activator (36). Foxp3-Cre mediated depletion of Rel-A in established Treg cells resulted in defective effector Treg cells that led to the development of an autoimmune syndrome characterized by a massive T cell activation, immune infiltrations of several tissues, as well as the production of inflammatory cytokines, and autoantibodies (36, 37). Furthermore, Rel-A deficient Treg cells were unstable and lost *Foxp3* expression becoming ex-Tregs expressing high amounts of proinflammatory cytokines IFNγ and TNFα (36).

*Bcl10* is a gene in the Carma1-Bcl10-Malt1 (CBM) signaling complex that controls NF-kB and MAPK activation in T cells following TCR activation (38). Bcl10 is necessary for the development of Treg cells and their suppressive function. *Bcl10*-deficient Treg cells converted in proinflammatory effector T cells secreting IFNγ, leading to a fatal systemic autoimmunity (39). This indicated that Bcl10-mediated NF-kB activation is required for Treg cell development and function. Previous studies have reported that HIF1-α directly binds to the IFNγ promoter in VHL-deficient Treg cells, a model described later in the text, provoking an increased IFNγ production and impairing Treg cell function (40). This phenotype is also displayed in *Bcl10*-deficient Treg cells (39).

Sclerostin domain-containing protein 1 (SOSTDC1) is selectively expressed in Tfh cells (41), which secretes this factor once they have lost the ability to help GC B cells (42). SOSTDC1 deficiency greatly reduced the generation Tfr cells, which in turn enhanced humoral immunity against viruses (42). Mechanistically, SOSTDC1 inhibits the canonical WNT-β-catenin pathway (43), which in turn inhibits Treg cell differentiation (44, 45). It should be noted that an autoimmune phenotype was not reported in these mice. This implies that although the differentiation of tTreg cells into Tfr cells was impaired, Treg cells themselves were functional and the effect of SOSTDC1 secreted by Tfh cells is confined to the GCs. Inhibition of Tfr cell differentiation in SOSTDC1-deficient mice was mediated by the stabilization of β-catenin (42). As a negative feedback loop, late-stage Tfh cells secrete SOSTDC1, which commits Treg cells in the GC to the Tfr fate by blocking WNT stimuli. Uncontrolled WNT-β-catenin signaling plays a role in autoimmune diseases (46), which may be due, at least in part, to defective Treg and Tfr cell differentiation.

## Genes Regulating Treg Cell Function and Stability Through Their Metabolism

Mammalian target of rapamycin corresponds to two kinase complexes, mTORC1 and mTORC2, which function as a central metabolic checkpoint. The functional links between metabolism and effector functions has been dissected in T cells, in which the integration by mTOR of the stimulatory signals and the energy status of the cells plays a critical role (47). Treg cells display diminished activity of the mTOR pathway as compared to Teff cells (46, 47), and increased mTOR activity negatively affects the generation and function of Treg cells (48–51). However, mTORC1 deficiency profoundly impairs Treg development and function (52). Mechanistically, mTORC1 enables cholesterol synthesis and lipid metabolism that are triggered by IL-2 signaling, both for which being required for Treg cell proliferation and the upregulation of suppressive molecules. mTOR signaling is required for the generation and function of both tTregs and pTregs, and its Foxp3-driven deletion impairs mitochondrial metabolism and oxidative phosphorylation, which is the main source of energy in Treg cells (53). Accordingly, Treg-specific deletion of the mitochondrial transcription factor *Tfam* severely impaired Treg suppressive functions (53). A recent genome-wide CRISPR/Cas9 screen combined with *in silico* analyses of protein-protein interaction networks identified novel regulatory modules that mediate mTORC1 signaling in Treg cells (54). The requirement for the expression of *Sec31a* and *Ccdc101*, two key genes in these modules, was validated when their deficiency in Treg cells impaired their suppressive functions and led to inflammatory phenotypes. SEC31A promotes mTORC1 activation by interacting with the GATOR2 component SEC13 to protect it from SKP1-dependent proteasomal degradation. Therefore, SEC31A expression is necessary to maintain mTORC1 activation in Treg cells. On the other hand, CCDC101 is a member of the SAGA complex, a potent inhibitor of mTORC1. Therefore, CCDC101 limits the expression of glucose and amino acid transporters and maintains a relative metabolic quiescence that characterizes Treg cells. *Ccdc101-*deficiency impairs Treg cells by unleashing an overreactive mTORC1. Additionally, Lamtor1, a lysosomal scaffold protein for mTORC1 is also important for Treg cell survival. Mice with Lamtor1-deficient Treg cells develop severe autoimmunity showing that Lamtor1 is a vital intrinsic factor for Treg suppressive functions, but not for their development and survival (55).

PP2A is a serine-threonine phosphatase composed of a catalytic C subunit PP2A_c_, a scaffold A subunit PP2A_A_ and a regulatory B subunit PP2A_B_ (56). PP2A is highly expressed in Treg cells, and mice with a Treg-specific deletion of a member of the PP2A_A_ subunit developed multi-organ autoimmunity with similarities to the scurfy phenotype (57). This indicated that PP2A activity is required to maintain Treg cells. PP2A_A_-deficiency increased mTORC1 activity in Treg cells, resulting in enhanced glycolysis and oxidative phosphorylation (57), a phenotype that was reversed by a treatment with mTOR inhibitor rapamycin. Therefore, PP2A activity is necessary to prevent mTORc1 overactivation, a process essential for suppressive function of Treg cells. In addition, PP2A_c_ is required for Treg cell to function by preventing the loss of expression of the IL-2Rβ chain, enabling IL-2 signaling (58). PPP2R2D is a regulatory subunit of PP2A whose expression is increased in T cells from patients with SLE. Mice with PPP2R2D-deficient T cells developed a reduced systemic autoimmunity in response to TLR7 activation (59). Furthermore, PPP2R2D-deficiency enhanced the suppressive function of Treg cells, which was supported by an increased IL-2 transcription in conventional T cells, a process that is negatively regulated by PPP2R2D (59). Therefore, PPP2R2D regulates Treg cells through PP2A in a cell-extrinsic manner (IL-2 secretion from conventional T cells), as opposed to PP2A controlling Treg function through mTORC1 in a cell-intrinsic manner.

HIF-1α and HIF-2α are two master transcription factors responsible for the physiological responses to hypoxia (60). Under normoxic conditions, prolyl hydroxylase domain proteins (PHD2/PHD3) hydroxylate HIF-1α and HIF-2α allowing for their recognition by von-Hippel Lindau tumor suppressor (VHL)-containing E3 complex, ubiquinating the transcription factors for proteasomal degradation. This process is interrupted under hypoxic conditions, allowing the accumulation of HIF-1α and HIF-2α (61). In immune cells under normoxic conditions, the expression of HIF-1α can also be increased by mTOR activation (62) and induce glycolysis (63). Germline *Hif1a*-deficiency promoted the differentiation of Treg cells over Th17 cells (64, 65). Mechanistically, HIF-1α promotes Foxp3 degradation by the proteasome (64). Germline *Hif1a* deficiency also inhibited glycolysis in favor of mitochondrial metabolism, which promoted Treg cell differentiation (65). Interestingly, *Hif1a*-deficiency in established Treg cells (through Foxp3-Cre mediated deletion) did not impair Treg cell function (66). This indicated that HIF-1α regulates Treg cell differentiation but not their maintenance and function. *Hif2a*-deficiency in established Treg cells impaired their suppressive activity despite normal *Foxp3* expression (66). Moreover, *Hif2a*-deficient Treg cells showed an enhanced secretion of IL-17 (66). Importantly, patients with SLE and associated lupus nephritis have increased numbers of IL-17-producing Treg cells in their peripheral blood (67). These studies demonstrate a complex crosstalk between HIF-1α and HIF-2α in Treg cells in which HIF-1α prevents their differentiation and HIF-2α stabilizes their function.

VHL-deficiency in Treg cells impaired their suppressive activity and stability leading to massive inflammation (40). VHL-deletion induced a HIF-1α-mediated expression of glycolytic enzymes in Treg cells that promoted Th1 differentiation. Moreover, HIF-1α directly activates the *Ifng* promoter. These results contrast with the lack of phenotype resulting from direct deletion of *Hif1a* in Treg cells (59), and suggest that HIF-2α constitutive expression in VHL-deficient Treg cells is likely to play a role.

Serine/Arginine-rich splicing factor 1 (SRSF1) is the prototype member of the highly conserved serine 1 arginine (SR) family of RNA-binding proteins (68). SRSF1 expression was decreased in the T cells of SLE patients with severe disease showing an overactive T cell phenotype (69). Deletion of SRSF1 in T cells led to systemic autoimmunity and lupus nephritis that was associated with mTOR activation in T cells (70). Treg-specific deletion of SRSF1 also led to systemic autoimmunity with Treg cells losing their suppressive function and producing proinflammatory cytokines (70). As with pan-T cell deletion, SRSF1-deficient Tregs displayed a highly glycolytic metabolism and mTOR activation.

## Treg Cell Regulation in Spontaneous Mouse Models of Lupus

Many studies have documented alterations in Treg numbers and functions in spontaneous mouse models of lupus (71). Multiple mechanisms are responsible for these phenotypes, with a major contribution of the inflammatory milieu created by cytokines such as Type 1 IFN and IL-6. Whether the genetic susceptibility that drives lupus pathogenesis in these models affects intrinsically Treg cells, at least in part, is less understood. The frequency of Treg cells varies across a wide range in mice and humans without pathogenic consequences (72). NZW mice do not develop autoimmunity, but their genome contains lupus susceptibility genes that are revealed when combined with other genomes such as NZB or BXSB (73). NZW mice present a low frequency of Treg cells, which was found to be cell-intrinsic and due to a low Foxp3 expression leading to a poor stability of the Treg program (72). Although NZW Treg cells express a distinctive transcriptional profile, it could not be attributed to a single genetic defect. Therefore, the NZW Treg phenotype is likely to be supported by a complex polygenic inheritance, similar to lupus susceptibility as a whole in NZW-derived strains (74). However, we propose that these intrinsically defective NZW Treg cells become pathogenic when combined with other immune defects induced by alleles from lupus-prone strains.

The (NZB x NZW) F1-derived NZM2410 strain is a model of lupus in which an analysis of genetic susceptibility has been conducted, and genes regulating T cell function have been identified (74). NZM2410 mice carry three major susceptibility loci associated with lupus nephritis, *Sle1*, *Sle2*, and *Sle3* (75). Congenic strains carrying separately each of these loci on a non-autoimmune C57BL/6 (B6) background present distinct autoimmune endophenotypes that correspond in combination to the lupus phenotype of the parental strain (76). *Sle1* had the strongest linkage to lupus nephritis and its expression is necessary for the development of autoimmunity in NZM2410 mice (77). *Sle1* regulates the function of T cells (78) in a cell-intrinsic manner (79), and it decreases the number and function of Treg cells (78). *Sle1* corresponds to at least three sub-loci, *Sle1a, Sle1b*, and *Sle1c* (80). Within *Sle1a*, genetic linkage analysis identified an interacting locus *Sle1a1* responsible for expanding the number of activated CD4^+^ T cells while reducing the frequency of pTreg cells (81). *Sle1a1* only contains one functional gene, *Pbx1* (81), a transcription factor required for mammalian organogenesis (82). Pbx1 is required for the development of B cells and the function of hematopoietic stem cells (83, 84), but its function in T cells had not been characterized. *Sle1a1* corresponds to the overexpression of the truncated splice isoform Pbx1-d over Pbx1-b, the normal isoform, in T cells (85). Pbx1-d lacks both the DNA-binding and HOX-binding domains and functions as a dominant negative (86). The mouse and human PBX1 proteins share complete homology, and PBX1-D was found more frequently in the CD4^+^ T cells from SLE patients than healthy controls (85). Furthermore, PBX1-D expression in human CD4+ T cells is associated with defective Treg cells (87). Mice overexpressing Pbx1-d in T cells replicated the phenotypes of B6.*Sle1a1* congenic mice as previously mentioned (88). Pbx1-d transgenic overexpression in T cells impaired iTreg differentiation as well as the induction or maintenance of pTreg cells in a cell-intrinsic manner (88). On the other hand, Pbx1-d overexpression in CD4^+^ T cells expanded Tfh cell differentiation (88). These results suggest that Pbx1 regulates the balance between Treg and Tfh cells, and that Pbx1-d contributes to autoimmunity by tilting the balance in favor of Tfh over Treg cells. This impaired Pbx1-d-mediated T cell homeostasis has consequences on lupus associated atherosclerosis, with chimeric atherosclerosis-prone mice carrying Pbx1-d expressing T cells developing more severe lesions than mice carrying Pbx1-b expressing T cells (89). Furthermore, there is evidence that dyslipidemia and Pbx1-d expression synergized to impair Treg cell functions. The mechanism by which Pbx1 and its dominant negative Pbx1-d isoform regulate T cell function has not been established yet. Interestingly, Pbx1 directly upregulates NFIL3 expression (90), and NFIL3 regulates the expression of Foxp3 and other Treg-associated genes (29). A disruption of the Pbx1/NFIL3 axis is therefore a potential mechanism by which Pbx1-d may alter the Treg/Tfh cell balance in favor of autoimmunity.

Within the *Sle1c* locus (91), recombinant congenic analysis mapped an activated CD4^+^ T cell phenotype to the *Sle1c2* sub-locus and the estrogen-related receptor gamma (*Esrrg*) gene it contains (91). *Esrrg* is essential in maintaining mitochondrial metabolism through activation of oxidative phosphorylation, the electron transport chain and ATP production in multiple cell types (91), but its function in T cells was unknown. *Esrrg* expression is reduced in the CD4^+^ T cells of B6.*Sle1c2* congenic mice, in association with altered mitochondrial functions and a decreased mitochondrial mass (91). This phenotype is consistent with that of CD4^+^ T cells of SLE patients in which mitochondrial defects have been described (92). *Esrrg* deletion in Treg cells altered the expression of genes involved in mitochondrial and Treg programs (93). This led to impaired suppressive function as well as differentiation into Tfr cells, which allowed for greater Tfh cell and humoral responses. These results suggest that the hypomorph *Esrrg* lupus susceptibility allele contributes to autoimmune pathogenesis by reducing the metabolic fitness of Treg cells.

## Conclusion

In summary, several genes have been identified as being responsible for sustaining the differentiation, function, and stability of Treg cells. The most common approach has been reverse genetics. Only a few Treg-specific studies have been conducted, but continued analyses of selective gene knockouts or overexpression models could advance our knowledge of novel genes that negatively or positively control Treg cells. However, CRISPR/Cas9 screens such as the one recently performed for mTORC1 activation in Treg cells (54) are likely to accelerate the speed of discovery and uncover novel genetic pathways through a less biased evaluation than classical reverse genetic approaches. The dissection of genetic susceptibility in a spontaneous mouse model of lupus has identified two genes that directly impact Treg cell homeostasis. So far, genetic loci associated with human lupus susceptibility, or susceptibility to other autoimmune diseases, have not been linked with Treg phenotypes. It is therefore unknown if allelic variations directly impacting Treg phenotypes confer autoimmune susceptibility in human populations.

**Figure 1:**
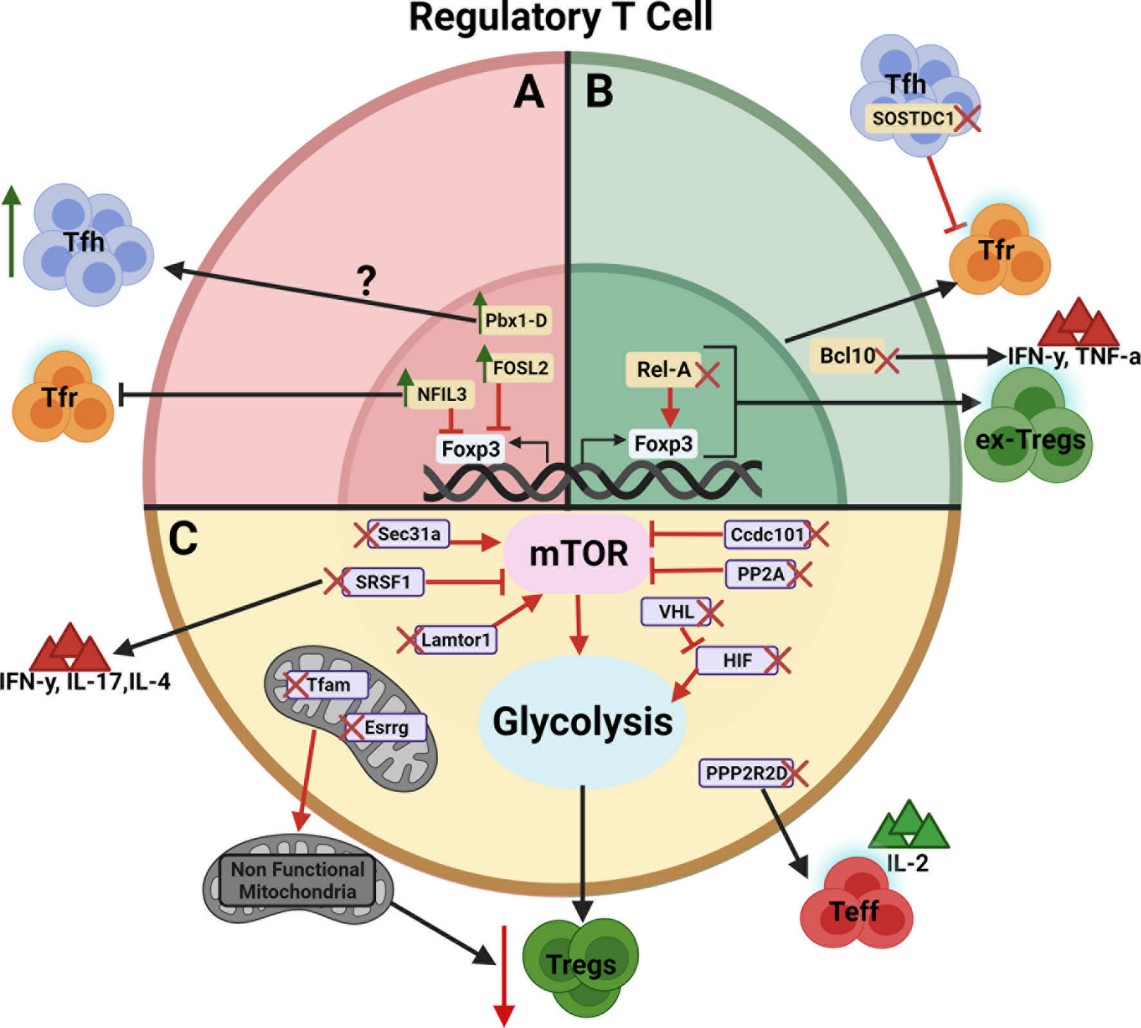
Schematic view of genes regulating Treg cell development, function, and/or stability. The genes are presented according to their effect on Treg cells. **(A)** Negative regulators whose over expression leads to an expansion of Tfh cells or inhibition of Tfr cells (Green arrows indicates gene overexpression).? indicates that the Pbx1-d direct target is unknown. **(B)** Positive regulators whose deletion leads to the inhibition of Tfr cells or the generation of ex-Treg cells producing IFNγ and TNFα (Red X indicates gene deletion). **(C)** Genes regulating Treg cells through their metabolism by way of mTOR, glycolysis and/or mitochondria metabolism leading to decreased immunosuppressive activity. Red arrows between genes and their target indicate an enhancing effect with expression of the target being decreased by the gene deletion. Red blocked arrows indicate an inhibitory effect with expression of the target being increased by the gene overexpression. Figure created with BioRender.com.

The majority of genes that have been identified to regulate Treg cells either directly control Foxp3 expression or their cellular metabolism (**Figure 1**). Treg cells are highly sensitive to mTOR activation, requiring “just the right amount” for optimal differentiation and suppressive function. Several genes have been identified in mice to maintain this “Goldilocks” homeostasis. The maintenance of mitochondrial metabolism or glycolysis, which is partially under mTORC1 control, is also required by Treg cells. It is predicted that other metabolic genes are also involved, and *in silico* analyses of protein-protein networks may be useful in pinpointing critical nodes in these networks.

Adoptive Treg cell therapies are being evaluated in clinical trials for autoimmune diseases and transplantation (94). The identification of regulatory networks that ensure their stability hand functions has great translational potentials to maximize these approaches. This knowledge could also benefit efforts to deactivate Treg cells in the tumor microenvironment to potentiate immunotherapies. This will require a comprehensive validation of these genetic pathways in human Treg cells, although the restraints of the read-out to *in vitro* suppression greatly limit the scope and the interpretation of these translation studies.

## Author Contributions

TR and LM wrote the review. All authors contributed to the article and approved the submitted version.

## Funding

This publication is supported by a grant from the NIH R01 AI045050 to LM

## Conflict of Interest

The authors declare that the research was conducted in the absence of any commercial or financial relationships that could be construed as a potential conflict of interest.

## Publisher’s Note

All claims expressed in this article are solely those of the authors and do not necessarily represent those of their affiliated organizations, or those of the publisher, the editors and the reviewers. Any product that may be evaluated in this article, or claim that may be made by its manufacturer, is not guaranteed or endorsed by the publisher.
